# Quantitative myocardial mapping using 3.0 T cardiac magnetic resonance imaging in dogs: adenosine stress–rest evaluation

**DOI:** 10.3389/fvets.2026.1850159

**Published:** 2026-07-15

**Authors:** Chang-eun Lee, Kichang Lee, Hakyoung Yoon

**Affiliations:** Department of Veterinary Medical Imaging, College of Veterinary Medicine, Jeonbuk National University, Iksan-si, Republic of Korea

**Keywords:** canine, cardiovascular magnetic resonance, extracellular volume, T1 mapping, T2 mapping

## Abstract

High-field cardiac magnetic resonance imaging (MRI) enables noninvasive assessment of myocardial tissue characteristics in anesthetized dogs; however, adenosine stress myocardial mapping at 3.0 T remains insufficiently characterized. This study evaluated stress–rest changes and level-dependent variation in quantitative myocardial mapping indices. Seven clinically asymptomatic dogs not receiving cardiac medications underwent 3.0 T cardiac MRI under general anesthesia in this prospective observational methodological study. Each dog completed two left ventricular myocardial mapping sessions (adenosine stress and rest) separated by ≥48 h. During stress, adenosine was infused at 140 μg/kg/min. T1 native, T1 post, T2, and extracellular volume fraction (ECV) were acquired at basal, mid, and apical short-axis levels. Stress–rest differences were tested with the Wilcoxon signed-rank test, and basal–mid–apical differences were assessed using generalized estimating equations (GEE) (*p* < 0.05). Under adenosine stress, global T1 native and T2 were 6.73 and 10.16% higher than at rest, whereas global T1 post and ECV did not change significantly. Level-wise analyses showed basal predominance for T1 native, a modest stress-only basal–mid difference for ECV, and lower basal T2 at rest. At 3.0 T, adenosine stress was associated with measurable increases in myocardial T1 native and T2 in anesthetized dogs, supporting further evaluation of protocol-specific, stress-associated mapping responses without relying solely on contrast-enhanced indices. Because mapping values varied across basal, mid, and apical levels, myocardial sampling level should be considered when interpreting or comparing canine stress–rest maps.

## 1 Introduction

With increasing longevity in both human and veterinary populations, the global burden of cardiovascular disease continues to rise, and advances in diagnostic imaging have played a major role in early detection and characterization (1–3). In human cardiology, advanced imaging modalities, including cardiovascular magnetic resonance (CMR), are routinely used to evaluate cardiac anatomy, perfusion, and myocardial tissue properties within standardized diagnostic frameworks (1, 2). In veterinary practice, transthoracic echocardiography (TTE) remains the principal cardiac imaging modality due to its accessibility and real-time, repeatable nature (3, 4). However, its dependence on operator expertise and acoustic windows limits the detection of subtle or diffuse myocardial abnormalities, particularly at subclinical stages (3, 4). Consequently, modalities long established in human medicine, such as CMR, are increasingly being adopted in small-animal cardiology (5). Yet, direct application of human CMR standards to dogs is inappropriate. Species-specific factors, together with the field-strength dependence of mapping indices, require veterinary-specific considerations (3, 5, 6).

Among advanced cardiac imaging modalities, CMR enables comprehensive cardiac phenotyping within a single examination. It integrates ventricular morphology, quantitative functional assessment, myocardial tissue characterization, as well as perfusion and flow assessment (4, 6, 7). At 3.0 T MRI, higher signal-to-noise and contrast-to-noise ratios can improve spatial or temporal resolution or shorten acquisition times (3, 6). Standardized acquisition and analysis are therefore important for quantitative mapping (4). Myocardial mapping provides quantitative tissue indices that can detect diffuse myocardial abnormalities often missed by conventional imaging, thereby strengthening tissue characterization beyond morphology alone (4). T1 native, T1 post, T2, and extracellular volume fraction (ECV) reflect myocardial water content and interstitial expansion and can support assessment of diffuse myocardial change (4, 8). In addition to static indices, stress–rest reactivity under pharmacologic vasodilation can be quantified with mapping (9).

In dogs, myocardial tissue characterization has received increasing attention, particularly in chronic mitral valve disease due to myxomatous degeneration, which is the most common cardiovascular disorder and a leading cause of congestive heart failure (10). Current clinical classification and therapeutic decision-making in canine myxomatous mitral valve disease rely primarily on clinical signs, cardiac remodeling, and echocardiographic criteria, which are essential for staging but provide limited direct information about diffuse myocardial tissue injury or microvascular dysfunction. Necropsy and pathology studies have reported myocardial lesions, including necrosis and fibrosis, even in clinically healthy animals, suggesting that subclinical myocardial injury and remodeling are not uncommon (11, 12). In dogs with congestive heart failure secondary to myxomatous mitral valve disease, myocardial fibrosis and small-vessel arteriosclerosis have been described and may parallel increases in circulating cardiac troponin I (13). Therefore, noninvasive myocardial tissue characterization may provide complementary information beyond valve morphology and chamber remodeling, although its therapeutic implications, management relevance, and prognostic value in dogs remain to be established in future disease-specific studies.

The adenosine stress–rest protocol uses a pharmacologic vasodilator to transiently increase coronary blood flow and myocardial blood volume, thereby probing coronary microcirculatory function and myocardial physiologic reserve (6). This stress component is relevant to myocardial disease because small-vessel arteriosclerosis or microvascular dysfunction may not be apparent from resting morphology alone, whereas stress reactivity can test the functional reserve of the coronary microcirculation. Adenosine exerts its effects predominantly via adenosine A2A receptor–mediated vasodilation and has a long-standing role in noninvasive ischemia testing (14). In mapping, stress–rest comparison of T1 native and T2 can capture stress-associated myocardial responses (9). In human studies, non-ischemic myocardium shows a modest increase in T1 native and T2 during stress, whereas ischemic or infarcted regions may show blunted or absent reactivity (9). In veterinary cardiac imaging, prior canine adenosine stress CMR has been reported using dynamic contrast-enhanced perfusion imaging; however, stress–rest myocardial T1/T2 or ECV mapping at 3.0 T remains insufficiently characterized in dogs (6, 15, 16).

For stress–rest mapping to be interpretable in future canine disease cohorts, baseline level-wise variation must also be defined. Regional analysis across basal, mid, and apical levels is biologically motivated. The left ventricle shows base-to-apex variation in architecture, and coronary perfusion territories do not map uniformly onto ventricular geometry (17, 18). In healthy humans at 3.0 T, mapping studies have demonstrated small but reproducible slice- and segment-level variability under standardized protocols (19, 20). In dogs, recent 1.5 T studies have similarly shown good reproducibility and documented slice- and sequence-dependent differences, including a base-to-apex increase in T2 without consistent slice-wise differences in T1 native or ECV (21). Therefore, clinically meaningful application of canine stress–rest mapping requires baseline protocol-specific information not only on global stress-associated changes but also on basal–mid–apical variability.

This prospective observational methodological study used 3.0 T CMR to characterize baseline protocol-specific adenosine stress–rest differences in T1 native, T1 post, T2, and ECV in anesthetized clinically asymptomatic dogs and to evaluate basal–mid–apical variability in these mapping indices. It was hypothesized that adenosine stress would be associated with increases in T1 native and T2, whereas level-dependent differences would be present across selected mapping parameters.

## 2 Materials and methods

### 2.1 Study design and animals

This prospective observational methodological study was approved by the Institutional Animal Care and Use Committee (IACUC approval No. JBNU NON2025-059). Client-owned dogs were prospectively enrolled by convenience sampling at Jeonbuk National University with written owner consent. Enrollment was limited to clinically asymptomatic dogs that were not receiving cardiac medications and that met predefined anesthetic safety criteria based on comprehensive preanesthetic screening. Before anesthesia, all dogs underwent physical examination, complete blood count, serum biochemistry, electrolyte analysis, thoracic radiography, and transthoracic echocardiography. Each dog underwent two mapping sessions, rest and adenosine stress, separated by at least 48 h.

Primary inclusion criteria were absence of cardiovascular clinical signs, no current use of cardiac medications, and acceptable anesthetic risk based on preanesthetic evaluation. Mitral regurgitation (MR) and tricuspid regurgitation (TR) were identified and graded using preanesthetic transthoracic echocardiography. Dogs with MR were eligible only when no structural cardiac remodeling was present and the findings were consistent with American College of Veterinary Internal Medicine stage B1 myxomatous mitral valve disease or lower-risk subclinical disease status (10). Dogs with TR were included only when regurgitation was mild, defined by peak TR velocity <3.0 m/s and absence of echocardiographic indicators of pulmonary hypertension.

For myocardial mapping analysis, dogs were required to have normal global systolic function on transthoracic echocardiography, no evidence of concentric or eccentric ventricular remodeling, no first-pass perfusion defect, and no late gadolinium enhancement on CMR. Dogs were excluded if they had moderate-to-severe valvular regurgitation, pulmonary hypertension, clinically relevant arrhythmia, imaging evidence of myocardial ischemia or replacement fibrosis, or any systemic condition considered to increase anesthetic risk or substantially affect cardiovascular interpretation.

### 2.2 Anesthesia and monitoring

Dogs were premedicated with butorphanol (0.2 mg/kg IV). Anesthesia was induced with propofol (4–6 mg/kg IV to effect), followed by endotracheal intubation and maintenance with isoflurane (1.0–1.5%) in 100% oxygen under mechanical ventilation. Heart rate, oxygen saturation, end-tidal CO₂, and noninvasive blood pressure were monitored. Arterial blood gas variables, including pH, arterial oxygen tension, arterial carbon dioxide tension, and lactate, were not measured. Normal saline was administered at 2.5–5.0 mL/kg/h, and active warming was applied to maintain normothermia. If hypotension or bradycardia occurred, initial management consisted of adjusting anesthetic depth and crystalloid infusion rate; anticholinergic agents were administered only when clinically necessary.

### 2.3 MRI system and scout workflow

All examinations were performed on a 3.0 T MRI system (uMR 780, United Imaging Healthcare, Shanghai, China). Dogs were positioned in dorsal recumbency. A 32-channel spine coil (dorsal) and a 12-channel superflex large coil (ventral) were used with electrocardiographic (ECG) gating.

**Figure 1:**
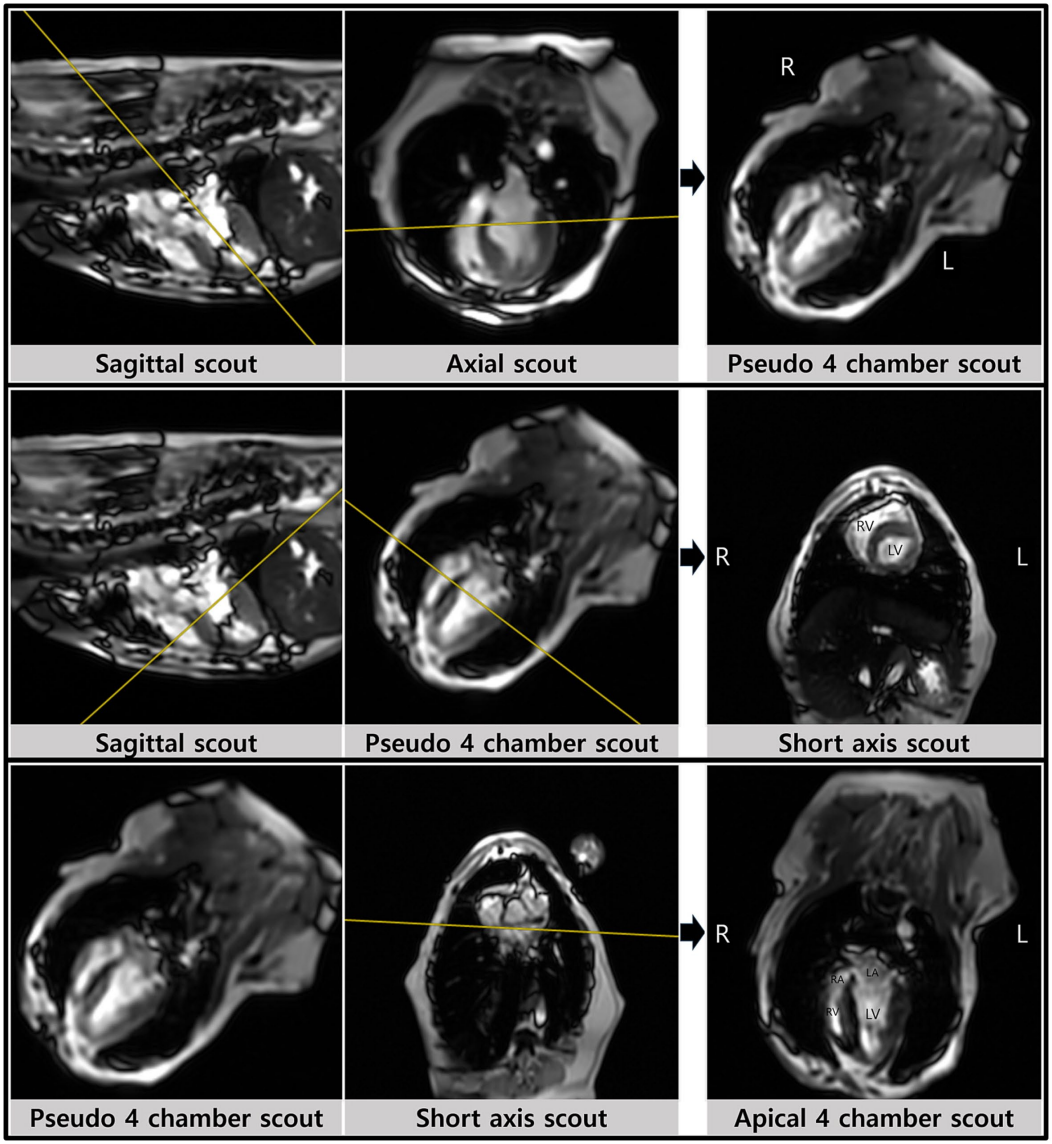
Stepwise slice orientation planning for cardiac magnetic resonance imaging (MRI). Sequential planning of imaging planes is illustrated from scout acquisition to final 4-chamber view. Top row: A pseudo 4-chamber plane was reconstructed using sagittal and axial scouts, with each reference line aligned along the left-ventricular (LV) long axis on the sagittal view and parallel (or perpendicular) to the LV long axis on the axial view. Middle row: A short-axis plane was planned perpendicular to the LV long axis as defined on both the sagittal and pseudo 4-chamber scouts. Bottom row: The final apical 4-chamber plane was derived from the pseudo 4-chamber plane and fine-tuned with reference to the short-axis plane to avoid crossing the LV outflow tract or aorta. Yellow lines indicate reference planes. R = right; L = left; LA = left atrium; LV = left ventricle; RA = right atrium; RV = right ventricle.

**Figure 2:**
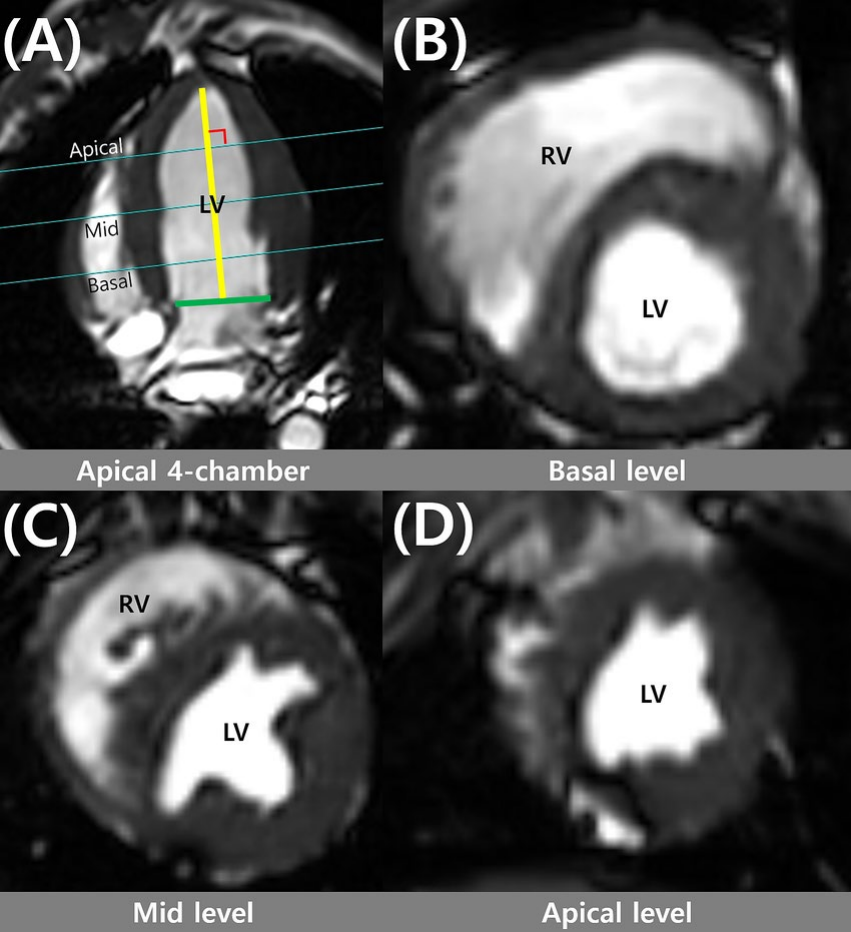
Slice orientation planning for short-axis level definition in cardiac magnetic resonance imaging (MRI). **(A)** Long-axis planning image corresponding to the apical 4-chamber view. The green line connects the hinge points of the mitral annulus, and the yellow line extends from its midpoint to the left-ventricular (LV) apex, defining the LV long axis. Short-axis planes for the basal, mid-ventricular, and apical levels were positioned perpendicular to this long axis at one-quarter intervals along the annulus-to-apex distance. **(B–D)** Representative short-axis planning images at the basal, mid-ventricular, and apical levels showing the left ventricle (LV) and right ventricle (RV).

**Figure 3:**
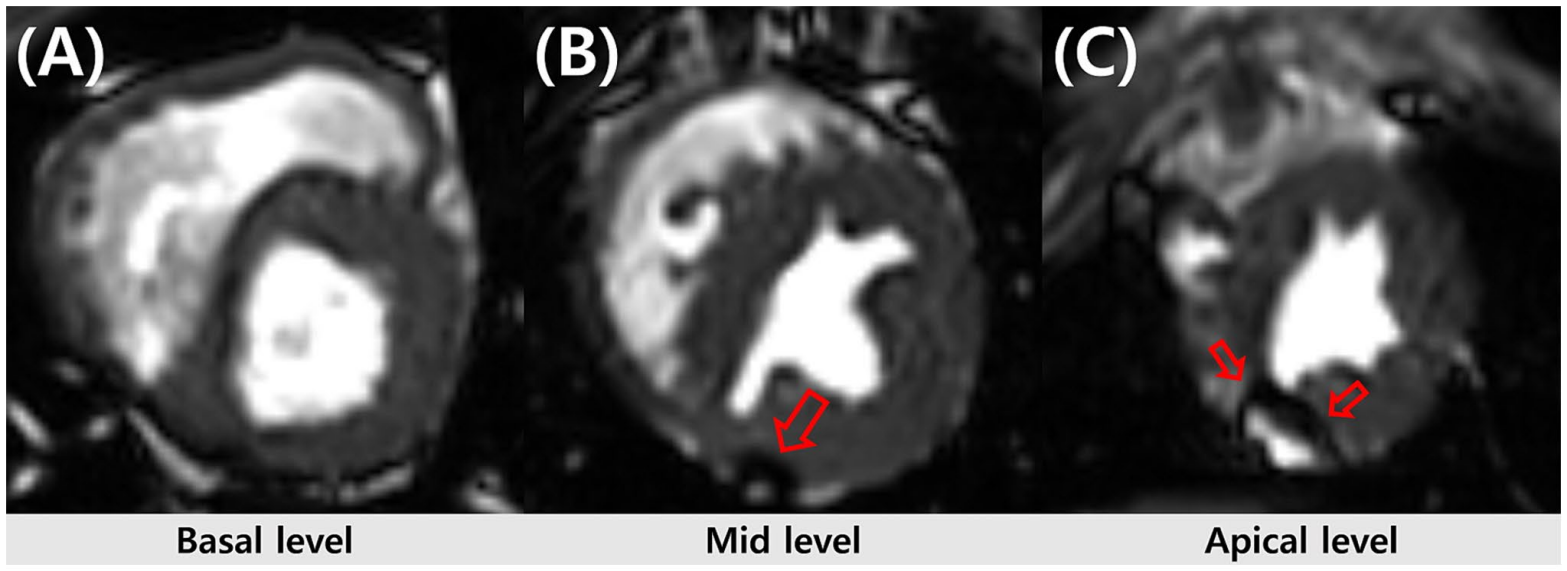
Slice-dependent off-resonance artifacts in bSSFP images despite optimized frequency-scout setting. Representative short-axis bSSFP images acquired at the basal **(A)**, mid **(B)**, and apical **(C)** levels at a center-frequency offset selected from the frequency scout (+50 Hz example). The basal slice shows uniform signal intensity without artifacts, whereas the mid and apical slices exhibit residual off-resonance banding (open red arrows). This finding illustrates that even with optimal frequency scouting, slice-dependent banding can occur at 3.0 T due to B₀ inhomogeneity and local susceptibility variation. This figure shows a representative artifact pattern and does not indicate that all mid- or apical-level segments were excluded; the full distribution of excluded segments is summarized in Supplementary Table S3. bSSFP, balanced steady-state free precession.

Basal, mid, and apical short-axis mapping levels were prescribed using localizers and a scout workflow (Figures 1, 2) (22). A balanced steady-state free precession (bSSFP) frequency scout was used to select a center frequency to minimize resonance banding artifacts, and the same heart-restricted, volume-selective shim settings were applied for subsequent mapping acquisitions (Figure 3).

### 2.4 Stress–rest design and injections

In the stress session, adenosine (Denosin injection; BCWorld Pharm. Co., Ltd.) was diluted in normal saline and infused intravenously at 140 μg/kg/min. This dose was selected based on prior canine 3.0 T adenosine stress perfusion CMR work and established vasodilator stress CMR protocols (16, 23). Adenosine infusion was maintained continuously, and T1 native and T2 mapping were initiated after at least 5 min of infusion to allow establishment of a stable pharmacologic stress condition. Heart rate and noninvasive blood pressure were monitored during adenosine infusion, but no predefined hemodynamic threshold was used as a formal stress-adequacy criterion. Adenosine infusion was continued throughout the stress session.

A contrast agent, gadoterate meglumine (Dotarem; Guerbet), was administered intravenously (0.1 mmol/kg, 2 mL/s), and first-pass perfusion imaging was acquired during injection. After 10 min, T1 post mapping and late gadolinium enhancement (LGE) imaging were acquired. The rest session followed the same protocol without adenosine.

### 2.5 Myocardial mapping acquisitions

T1 and T2 mapping were acquired (slice thickness 4.00 mm; field of view 300 × 300 mm^2^; flip angle 35°).

T1 mapping used a bSSFP-based modified Look–Locker inversion recovery sequence (MOLLI). T1 native was acquired with a 5(3)3 scheme and T1 post with a 4(1)3(1)2 scheme. Additional parameters were: matrix 179 × 256, repetition time 3.40 ms, echo time 1.61 ms, and bandwidth 1,200 Hz/pixel. No heart-rate correction was applied to the MOLLI-derived T1 values. Therefore, heart-rate dependency of MOLLI-based T1 mapping was considered during interpretation, particularly because heart rate varied among dogs and between stress and rest sessions (24).

T2 mapping used a T2-prepared bSSFP sequence with three preparation times (0, 30, and 55 ms). Additional parameters were: matrix 173 × 192, repetition time 3.48 ms, echo time 1.62 ms, and bandwidth 800 Hz/pixel.

### 2.6 Image analysis and ECV calculation

Three short-axis slices (basal, mid, and apical) were analyzed. Segmental analysis used the American Heart Association (AHA) 16-segment model (apical cap excluded) (17). Myocardial regions of interest (ROIs) were manually defined by a single observer (C. E. L.) as mid-myocardial rings while excluding the subendocardial and subepicardial borders to reduce partial-volume effects and boundary-related uncertainty. ROIs were positioned to avoid off-resonance bands whenever possible. Segments in which most of the myocardial area was affected by off-resonance banding, severe map-quality degradation, or contour misregistration were excluded from quantitative analysis and treated as missing values. In the raw segment-level dataset, excluded segments were assigned an analyzable ROI pixel count of zero and were not included in level-wise or global weighted averages. Pixel-proportion weighting indicates that each segment contributed to level-wise and global summary values in proportion to the number of myocardial pixels included in the final ROI; therefore, larger analyzable myocardial regions contributed more strongly, whereas excluded segments with zero pixels did not contribute to the weighted mean. All primary analyses were based on the first ROI measurement dataset. For intraobserver repeatability, the same observer repeated segment-level ROI delineation after a 5-month washout interval while blinded to the first measurements. The repeated analysis was performed using the same software and the same ROI placement and segment-exclusion criteria as the primary analysis. Repeated measurements were used only for intraobserver repeatability assessment.

ECV was calculated from paired myocardial and blood T1 values (pre- and post-contrast) and the pre-anesthesia hematocrit (Hct) using ΔR1: ECV(%) = (1 − Hct) × (ΔR1myo/ΔR1blood) × 100, where ΔR1 = (1/T1post − 1/T1pre) (4). Blood T1 was measured using an ROI placed in the LV cavity on the short-axis slice.

### 2.7 Statistical analysis

Normality of paired stress–rest differences was assessed using the Shapiro–Wilk test for global and level-wise mapping values. Because of the small sample size and non-normality in at least one paired comparison, within-dog stress–rest differences were evaluated using the Wilcoxon signed-rank test. Descriptive values are presented as mean ± SD in the main tables for continuity with the original quantitative summaries, while median [IQR] values and Shapiro–Wilk *p*-values for paired differences are provided in Supplementary Table S1. Figures display mean values or pairwise differences with 95% confidence intervals for visual interpretation. Standardized paired effect sizes were calculated for the primary global stress–rest endpoints as dz = mean *Δ*/SD Δ. Basal–mid–apical differences were assessed using generalized estimating equations (GEE) with dog as the clustering unit, an exchangeable working correlation structure, robust standard errors, and pixel-proportion weights at the segment level. Overall level effects were evaluated using Wald χ^2^ tests. Pre-specified pairwise contrasts among basal, mid, and apical levels were evaluated using robust Wald tests, and multiplicity was controlled using a stepwise family-wise error-rate procedure. Exploratory partial Spearman correlation analyses were performed to assess associations between heart rate and myocardial mapping parameters. These analyses were used to evaluate heart rate as a potential physiologic covariate rather than as a primary hypothesis test. Intraobserver repeatability was evaluated using segment-level paired measurements from the first and repeated ROI analyses. Segments with zero analyzable ROI pixels were excluded. Repeatability was summarized using intraclass correlation coefficients (ICC) from a two-way mixed-effects absolute-agreement model for single measurements and within-observer coefficients of variation (CV). The within-observer CV was calculated as the standard deviation of paired differences divided by √2 and expressed relative to the overall mean of the paired measurements. A two-sided *p*-value < 0.05 was considered statistically significant.

## 3 Results

### 3.1 Subjects and baseline characteristics

Seven client-owned dogs were included (Jindo, *n* = 1; Poodles, *n* = 3 [two miniatures, one toy]; Beagles, *n* = 3; age, 9–18 years; body weight 3.7–26 kg). All dogs demonstrated normal systolic function on transthoracic echocardiography, without evidence of concentric or eccentric remodeling. Two dogs had no echocardiographic abnormalities, whereas the remaining five had mild valvular regurgitation without clear myocardial remodeling: four had mild mitral regurgitation (MR) with concurrent mild tricuspid regurgitation (TR), and one had isolated mild tricuspid regurgitation. No abnormalities suggestive of myocardial ischemia were observed on first-pass perfusion imaging or LGE in any dog. Accordingly, the cohort was classified as clinically asymptomatic and non-ischemic, with near-normal/subclinical myocardial status suitable for protocol-specific quantitative mapping analysis. This cohort should not be interpreted as a strictly healthy reference population.

Mild elevations in serum hepatic enzyme activities were present in several dogs (Table 1), but these were interpreted as clinically stable non-cardiac comorbidities without evidence of hemodynamic relevance and were not considered exclusionary. Table 1 summarizes the baseline characteristics. Heart rate varied among dogs and between sessions. Stress HR ranged from 85 to 127 beats/min, whereas rest HR ranged from 69 to 111 beats/min (Table 1). Five dogs had higher HR during stress than during rest, while two dogs had lower HR during stress (Dog B, 86 vs. 111 beats/min; Dog G, 85 vs. 91 beats/min). In exploratory partial Spearman correlation analyses, HR was positively associated with T1 native (*r* = 0.675, *p* = 0.008) and T2 (*r* = 0.548, *p* = 0.043), but not with T1 post (*r* = −0.431, *p* = 0.124) or ECV (*r* = −0.218, *p* = 0.454) (Supplementary Table S2).

**Table 1:** Baseline characteristics of the dogs.

Dog ID	Sex	Age (year)	Body weight (kg)	Breed	Hct (%)	Stress HR (bpm)	Rest HR (bpm)	Stress BP (mmHg) [SYS/DIA (MAP)]	Rest BP (mmHg) [SYS/DIA (MAP)]	Clinical findings
A	Male	9	26	Jindo	55	123	99	100/65 (77)	115/75 (88)	ALT ↑154 (17–78); no MR/TR
B	Female	12	10.5	Beagle	44	86	111	118/78 (91)	130/88 (102)	ALT ↑123 (17–78); mild TR
C	Female	9	8.3	Beagle	48	122	69	92/55 (67)	90/52 (65)	No abnormal clinical pathology or echo findings
D	Female	10	7.8	Beagle	36	108	78	106/68 (81)	112/72 (85)	ALP ↑392 (47–254), ALT ↑136; mild MR, mild TR
E	Male	16	7.5	Miniature Poodle	53	121	75	88/50 (63)	95/55 (68)	Normal blood work; mild MR, mild TR
F	Male	10	3.7	Toy Poodle	47	127	75	90/52 (65)	104/62 (76)	ALP ↑405 (47–254); mild MR, mild TR
G	Male	18	4	Miniature Poodle	47	85	91	100/65 (77)	115/75 (88)	ALP ↑645 (47–254); mild MR, mild TR

Hct, hematocrit; HR, heart rate; BP, blood pressure; SYS, systolic; DIA, diastolic; MAP, mean arterial pressure; MR, mitral regurgitation; TR, tricuspid regurgitation; ALT, alanine aminotransferase; ALP, alkaline phosphatase.

### 3.2 Descriptive statistics

**Figure 4:**
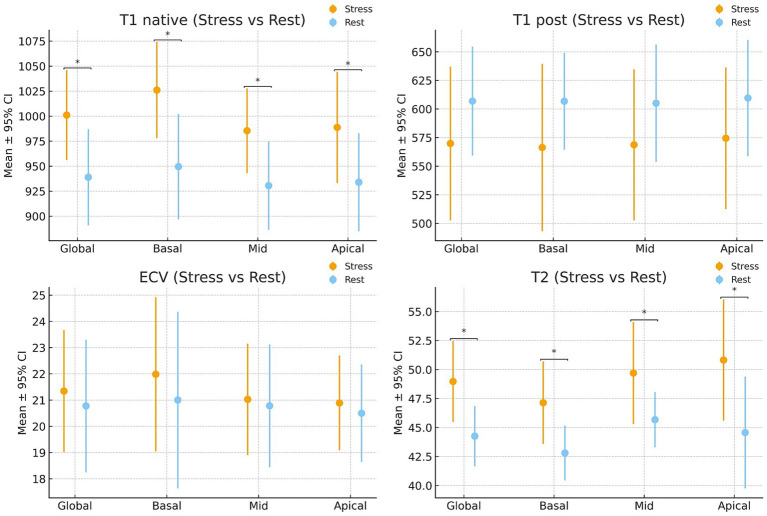
Stress–rest comparisons of global and regional myocardial mapping parameters (T1 native, T1 post, ECV, and T2). Global, basal, mid, and apical values of T1 native, T1 post, ECV, and T2 under adenosine stress and rest conditions. Data are shown as mean values with 95% confidence intervals across dogs (*n* = 7). * indicates *p* < 0.05 by Wilcoxon signed-rank test. ECV, extracellular volume fraction; CI, confidence interval.

Global and level-wise descriptive values under stress and rest conditions are presented as mean ± SD in Table 2. Figure 4 displays the corresponding stress–rest patterns using mean values with 95% confidence intervals. To align descriptive reporting with the non-parametric paired analyses, corresponding median [IQR] values and Shapiro–Wilk normality results for paired stress–rest differences are provided in Supplementary Table S1. The Shapiro–Wilk test indicated non-normality for the paired mid-level T1 native difference, supporting the use of the Wilcoxon signed-rank test in this small cohort.

**Table 2:** Global and regional (basal, mid, apical) mean values and stress–rest comparisons of myocardial mapping parameters (T1 native, T1 post, ECV, and T2).

Parameter	Global/level	Stress (Mean ± SD)	Rest (Mean ± SD)	Δ (Mean ± SD)	Δ % (Mean ± SD)	*p*-value
T1 native	Global	1001.18 ± 48.62	939.01 ± 51.93	62.17 ± 38.54	6.73 ± 4.38	<0.05*
T1 native	Basal	1026.11 ± 52.05	949.59 ± 56.92	76.53 ± 32.68	8.16 ± 3.67	<0.05*
T1 native	Mid	985.50 ± 45.75	930.60 ± 47.73	54.91 ± 44.80	6.03 ± 5.24	<0.05*
T1 native	Apical	988.84 ± 60.10	934.04 ± 52.96	54.80 ± 40.41	5.92 ± 4.46	<0.05*
T1 post	Global	569.85 ± 72.63	606.93 ± 51.42	−37.09 ± 90.77	−5.47 ± 15.47	0.297
T1 post	Basal	566.34 ± 79.14	606.80 ± 45.90	−40.46 ± 94.21	−6.12 ± 16.07	0.297
T1 post	Mid	568.66 ± 71.36	605.10 ± 55.46	−36.44 ± 91.81	−5.29 ± 15.58	0.297
T1 post	Apical	574.39 ± 66.92	609.56 ± 54.95	−35.17 ± 86.65	−5.08 ± 14.75	0.297
ECV	Global	21.22 ± 2.23	20.68 ± 2.50	0.54 ± 1.55	2.95 ± 7.82	0.578
ECV	Basal	21.67 ± 2.51	20.79 ± 3.10	0.88 ± 2.28	5.07 ± 11.50	0.578
ECV	Mid	21.03 ± 2.29	20.78 ± 2.53	0.25 ± 1.13	1.40 ± 5.54	0.938
ECV	Apical	20.89 ± 1.95	20.50 ± 2.00	0.39 ± 1.30	2.11 ± 6.65	0.578
T2	Global	48.97 ± 3.77	44.46 ± 2.69	4.51 ± 2.44	10.16 ± 5.44	<0.05*
T2	Basal	47.20 ± 3.77	42.80 ± 2.56	4.40 ± 3.36	10.38 ± 7.86	<0.05*
T2	Mid	49.69 ± 4.75	45.62 ± 2.58	4.07 ± 3.40	8.86 ± 7.18	<0.05*
T2	Apical	50.82 ± 5.65	45.45 ± 4.22	5.36 ± 4.67	12.07 ± 10.34	<0.05*

Values are mean ± SD across dogs (*n* = 7). Δ indicates the absolute difference (stress − rest). Δ%, percentage change relative to rest. Stress–rest *p*-values were calculated using the Wilcoxon signed-rank test. Corresponding median [IQR] values and Shapiro–Wilk *p*-values for paired stress–rest differences are provided in Supplementary Table S1. * indicates *p* < 0.05.

ECV, extracellular volume fraction; SD, standard deviation; IQR, interquartile range.

### 3.3 Image-quality exclusions and segment availability

Across all dogs, conditions, parameters, and myocardial segments, 896 parameter-specific segment observations were available for review. Of these, 23 segments (2.6%) were excluded from quantitative analysis because the final analyzable ROI pixel count was zero due to off-resonance banding, severe map-quality degradation, or contour misregistration. Exclusions were uncommon for T1 native (7/224, 3.1%), T1 post (1/224, 0.4%), and T2 (2/224, 0.9%), but were more frequent for ECV (13/224, 5.8%), particularly under the rest condition (11/112, 9.8%). By level, excluded observations accounted for 7/336 basal segments (2.1%), 9/336 mid-ventricular segments (2.7%), and 7/224 apical segments (3.1%). The distribution was therefore not confined to a single ventricular level, although the highest single-segment frequency occurred in the mid-inferolateral region. The parameter- and level-wise distribution of excluded segments is summarized in Supplementary Table S3.

### 3.4 Intraobserver repeatability

Intraobserver repeatability was assessed using repeated segment-level ROI measurements after a 5-month washout interval. Segments with zero analyzable ROI pixels were excluded from the paired analysis. Repeatability was high for all mapping parameters, with ICCs of 0.978 for T1 native, 0.984 for T1 post, 0.917 for ECV, and 0.962 for T2. Corresponding within-observer CVs were 1.0, 1.4, 3.6, and 2.2%, respectively (Supplementary Table S4).

### 3.5 Stress–rest comparisons

Stress–rest differences in myocardial mapping parameters are summarized in Table 2. At the global level, T1 native was higher during adenosine stress than at rest (1001.18 ± 48.62 ms vs. 939.01 ± 51.93 ms, *p* < 0.05). Similar stress-associated increases were observed at the basal, mid, and apical levels (all *p* < 0.05; Table 2).

In contrast, T1 post demonstrated a modest, non-significant decrease under stress (global *Δ* − 37.09 ± 90.77 ms; −5.47%, *p* = 0.297). ECV also exhibited only small, non-significant changes at the global and level-wise analyses (global Δ 0.54 ± 1.55 percentage points, 2.95%, *p* = 0.578; Table 2).

T2 was higher under stress at the global level (48.97 ± 3.77 ms vs. 44.46 ± 2.69 ms, *p* < 0.05), with concordant increases at the basal, mid, and apical levels (all *p* < 0.05; Table 2; Figure 4). Collectively, these findings indicate consistent stress-associated increases in T1 native and T2 across global and level-wise analyses, whereas T1 post and ECV remained relatively stable.

Exploratory paired effect-size estimates (Table 3) were used to contextualize the magnitude of the stress–rest differences descriptively. T1 native and T2 showed larger paired effect sizes (dz = 1.61 and 1.85, respectively) than T1 post (dz = −0.41) and ECV (dz = 0.35).

**Table 3:** Exploratory paired effect-size estimates for global stress–rest myocardial mapping differences.

Parameter	*n*	Mean Δ	SD Δ	Effect size (dz)
T1 native	7	62.17	38.54	1.61
T1 post	7	−37.09	90.77	−0.41
ECV	7	0.54	1.55	0.35
T2	7	4.51	2.44	1.85

Values represent paired global stress–rest differences aggregated at the dog level (*n* = 7). Effect size (Cohen’s dz) was computed as the mean paired difference divided by its standard deviation and was used as an exploratory descriptive estimate of effect magnitude.

ECV, extracellular volume fraction; dz, Cohen’s paired effect size; SD, standard deviation.

### 3.6 Level-wise and pairwise comparisons

Omnibus GEE analyses demonstrated significant level-wise effects for T1 native at both rest and stress (rest: Wald χ^2^ = 10.54, *p* = 0.005; stress: Wald χ^2^ = 18.871, *p* < 0.001), for ECV under stress (Wald χ^2^ = 12.856, *p* = 0.002), and for T2 under rest (Wald χ^2^ = 17.556, *p* < 0.001). No significant level-wise effects were detected for T1 post at either condition, ECV at rest, or T2 under stress (Table 4). For pairwise level comparisons, the *p*-values reported in Table 5 are adjusted *p*-values after multiplicity control for the three pre-specified contrasts within each parameter–condition model.

**Table 4:** GEE (Wald χ^2^) test results for myocardial mapping parameters under stress and rest conditions.

Parameter	Condition	Wald χ^2^ (df = 2)	*p*-value
T1 native	Rest	10.54	0.005**
T1 native	Stress	18.871	<0.001***
T1 post	Rest	0.595	0.743
T1 post	Stress	4.651	0.098
ECV	Rest	0.812	0.666
ECV	Stress	12.856	0.002**
T2	Rest	17.556	<0.001***
T2	Stress	2.942	0.230

*p*-values are from Wald χ^2^ tests using generalized estimating equations. * indicates *p* < 0.05. ** indicates *p* < 0.01. *** indicates *p* < 0.001.

ECV, extracellular volume fraction.

**Table 5:** Level-pairwise comparisons of myocardial mapping parameters (T1 native at rest and stress, ECV at stress, and T2 at rest) identified as significant in the overall Wald test.

Parameter	Condition	Contrast	Basal (Mean ± SD)	Mid (Mean ± SD)	Apical (Mean ± SD)	Δ Mean ± SD	Δ% Mean ± SD	*p*-value
T1 native	Rest	Basal-Mid	949.59 ± 56.92	930.60 ± 47.73		18.99 ± 22.84	2.02 ± 2.46	0.004**
T1 native	Rest	Basal-Apical	949.59 ± 56.92		934.04 ± 52.96	15.55 ± 27.20	1.68 ± 2.85	0.037*
T1 native	Rest	Mid-Apical		930.60 ± 47.73	934.04 ± 52.96	−3.44 ± 19.54	−0.32 ± 2.02	0.714
T1 native	Stress	Basal-Mid	1026.11 ± 52.05	985.50 ± 45.75		40.61 ± 26.87	4.13 ± 2.71	<0.001***
T1 native	Stress	Basal-Apical	1026.11 ± 52.05		988.84 ± 60.10	37.28 ± 37.39	3.88 ± 3.77	0.058
T1 native	Stress	Mid-Apical		985.50 ± 45.75	988.84 ± 60.10	−3.33 ± 36.47	−0.22 ± 3.56	0.491
ECV	Stress	Basal-Mid	21.67 ± 2.51	21.03 ± 2.29		0.65 ± 0.64	3.02 ± 3.16	0.009**
ECV	Stress	Basal-Apical	21.67 ± 2.51		20.89 ± 1.95	0.78 ± 0.93	3.62 ± 4.41	0.052
ECV	Stress	Mid-Apical		21.03 ± 2.29	20.89 ± 1.95	0.14 ± 0.73	0.59 ± 3.43	0.65
T2	Rest	Basal-Mid	42.80 ± 2.56	45.62 ± 2.58		−2.82 ± 2.42	−6.07 ± 5.01	<0.001***
T2	Rest	Basal-Apical	42.80 ± 2.56		45.45 ± 4.22	−2.65 ± 3.45	−5.38 ± 6.99	0.073
T2	Rest	Mid-Apical		45.62 ± 2.58	45.45 ± 4.22	0.16 ± 1.83	0.68 ± 4.15	0.822

Δ indicates the pairwise difference between myocardial levels (Basal–Mid, Basal–Apical, and Mid–Apical). Pairwise *p*-values were obtained from robust Wald tests after GEE modeling and adjusted for the three pre-specified pairwise contrasts within each parameter–condition model using a stepwise family-wise error-rate procedure. * indicates adjusted *p* < 0.05. ** indicates adjusted *p* < 0.01. *** indicates adjusted *p* < 0.001.

ECV, extracellular volume fraction; GEE, generalized estimating equations; SD, standard deviation.

**Figure 5:**
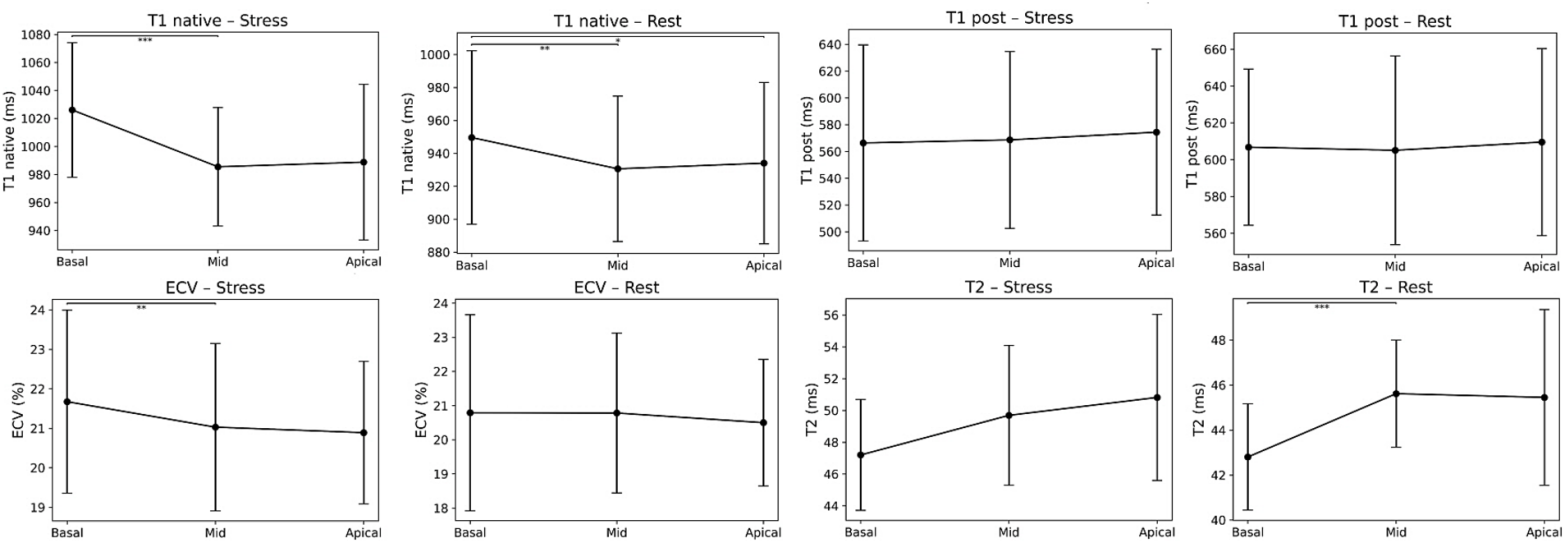
Level pairwise comparisons of myocardial mapping parameters. Segment-level pairwise differences for mapping parameters with significant level-wise effects. Error bars represent 95% confidence intervals; brackets indicate pairwise comparisons. * indicates adjusted *p* < 0.05. ** indicates adjusted *p* < 0.01. *** indicates adjusted *p* < 0.001. ECV, extracellular volume fraction; CI, confidence interval.

For T1 native, basal values were higher than mid values at both rest and stress (rest basal–mid: *Δ* = 18.99 ± 22.84 ms, Δ% = 2.02 ± 2.46%, *p* = 0.004; stress basal–mid: Δ = 40.61 ± 26.87 ms, Δ% = 4.13 ± 2.71%, *p* < 0.001). Basal values were also higher than apical values at rest (basal–apical: Δ = 15.55 ± 27.20 ms, Δ% = 1.68 ± 2.85%, *p* = 0.037) (Table 5; Figure 5).

Under stress, basal ECV was higher than mid ECV (Δ = 0.65 ± 0.64 percentage points, Δ% = 3.02 ± 3.16%, *p* = 0.009) (Table 5; Figure 5).

For T2 at rest, basal values were lower than mid values (basal–mid: Δ = −2.82 ± 2.42 ms, Δ% = −6.07 ± 5.01%, *p* < 0.001) (Table 5; Figure 5). All other pre-specified pairwise contrasts were not significant (Table 5; Figure 5).

## 4 Discussion

### 4.1 Summary of key findings

In stress–rest comparisons, T1 native increased at the global level (~6.7%), and T2 showed a similar global increase (~10.2%), with concordant changes across basal, mid, and apical levels (Table 2; Figure 4). By contrast, T1 post and ECV showed no significant stress–rest changes (Table 2). Level-wise analyses indicated a basal predominance for T1 native at rest and stress, a modest stress-only basal–mid difference for ECV, and a basal < mid/apical pattern for T2 at rest (Tables 4, 5; Figure 5).

### 4.2 Mechanisms of adenosine-induced stress and interpretation of results

Adenosine induces hyperemia primarily through A2A receptor–mediated vasodilation of the coronary microvasculature, leading to increased myocardial blood flow and blood volume (14, 25). In prior canine 3.0 T dynamic contrast-enhanced perfusion CMR, adenosine stress produced measurable stress–rest differences in myocardial perfusion parameters, supporting the physiologic plausibility of using adenosine as a vasodilator stressor in dogs (16). In the present study, adenosine dose, infusion timing, anesthetic management, and image-acquisition procedures were standardized across dogs. Therefore, the observed T1 native and T2 increases are best interpreted as protocol-specific stress-associated mapping responses, while inter-individual variation in vasodilator response should be considered expected biological variability.

T1 native is sensitive to hyperemia-related changes (including increased perfusion, blood volume, and free water content), making an increase during stress physiologically plausible. In this study, T1 native increased globally by approximately 6.7%, with a somewhat larger increase at the basal level (~8.2%) and smaller but concordant changes at the mid and apical levels (~6.0% and ~5.9%, respectively). The relatively greater basal increase should be interpreted cautiously. It may partly reflect true level-dependent physiologic or anatomic variation, but basal short-axis slices are also closer to the atrioventricular and aortic root planes and may be more vulnerable to through-plane motion, blood-pool contamination, and partial-volume effects. Because a fixed 4-mm slice thickness was used across dogs with a wide body-size range, the relative contribution of partial-volume effects may have differed among individuals. In addition, bSSFP-based mapping at 3.0 T remains susceptible to slice-dependent off-resonance effects, which can subtly alter signal sensitivity across levels (26, 27).

T1 post demonstrated little to no stress–rest difference, likely because the strong T1-shortening effect of gadolinium dominated over short-term hemodynamic changes. In addition, T1 post is highly sensitive to acquisition timing (i.e., time elapsed after contrast administration) and precise slice and cardiac-phase matching. Even small differences in post-contrast timing or subtle mismatches in slice position or cardiac phase during stress can alter myocardial and blood T1 values, thereby indirectly influencing ECV calculation (4, 28).

ECV reflects the structural extracellular fraction and is therefore expected to be relatively insensitive to acute vasodilation. In the present stress–rest protocol, ECV was included not as a primary marker of acute hyperemia, but as a structural comparator for interpreting stress-associated changes in T1 native and T2. The absence of a significant aggregated ECV difference between stress and rest is consistent with this role, because vasodilator stress is not expected to acutely change extracellular space fraction (4). However, ECV calculation depends on both myocardial and blood-pool T1 measurements and on accurate matching between native and post-contrast maps; therefore, regional ECV estimates may be more vulnerable to slice mismatch, contour misregistration, and blood-pool contamination than non-contrast T1 native or T2 values (4, 28).

Given its sensitivity to free water, edema, perfusion, and blood-volume changes, T2 is also expected to increase during vasodilator stress. In this study, T2 increased across all levels, with a global rise of approximately 10.2% and level-wise increases at the basal, mid, and apical levels. Experimental work further supports myocardial blood flow as an important determinant of adenosine-induced T2 changes, which is consistent with the direction of the present findings (29). Nevertheless, because direct myocardial blood flow was not measured, the observed T2 increase should be interpreted as an adenosine-associated mapping response rather than as a direct quantitative perfusion surrogate.

Overall, the stress–rest pattern across mapping parameters is compatible with a protocol-specific adenosine-associated response, particularly for T1 native and T2. Direct myocardial blood flow was not measured and no formal stress-adequacy threshold was applied; therefore, these changes should not be interpreted as quantitative perfusion surrogates or as proof of preserved vasodilator reserve in each dog. The larger paired effect sizes for T1 native and T2 compared with T1 post and ECV support the internal consistency of the main findings, although the magnitude of T1 native and T2 responses varied among dogs. Such dispersion is expected in a heterogeneous client-owned cohort and may reflect differences in body size, breed, age, heart rate, hematocrit, vascular tone, and adenosine responsiveness. Accordingly, the present findings should be interpreted as preliminary protocol-specific response patterns rather than narrow reference intervals.

### 4.3 Differences and interpretation across myocardial levels (basal–mid–apical)

T1 native values exhibited significant level-wise differences both at rest and during stress. At rest, basal values were significantly higher than mid and apical values, whereas the mid–apical difference was not significant. During stress, T1 native again followed a basal > mid ≈ apical pattern, indicating that level-dependent variation persisted across physiologic states. This basal predominance may reflect a combination of intrinsic anatomic–perfusion gradients, basal-plane proximity to the aortic root and atrioventricular junction, blood-pool contamination, partial-volume effects, and slice-wise technical sensitivity at 3.0 T (19, 26). Therefore, the basal pattern should not be interpreted as a purely physiologic gradient without considering acquisition geometry and body-size-related partial-volume effects.

T1 post values showed generally small and inconsistent level differences. The potent T1-shortening effect of gadolinium can obscure subtle level-dependent variations, which aligns with expectations for post-contrast indices (4).

ECV showed no differences across levels at rest, whereas during stress, the basal–mid comparison was the only statistically significant difference. The lack of significant basal–apical and mid–apical differences suggests a limited basal predominance rather than a consistent basal > mid > apical gradient. This pattern may reflect a combination of level-dependent blood-pool influence, basal-plane partial-volume effects, and the dependence of ECV on both myocardial and blood-pool T1 values rather than a true acute change in extracellular space fraction (4, 28).

T2 values demonstrated a significant basal < mid/apical pattern at rest, consistent with previous observations of slice-wise T2 variability in both human 3.0 T and canine mapping studies (21, 30). During stress, this level-wise difference was attenuated and no longer statistically significant. This may reflect a combination of adenosine-associated increases in myocardial blood volume and free water content across levels, together with the inherently greater variability of T2-prepared bSSFP mapping at 3.0 T.

More generally, these results indicate that level-wise variability in T1 native, ECV, and T2 likely reflects a mixture of true anatomic or perfusion-related variation along the ventricular long axis and technical factors associated with 3.0 T acquisition. Therefore, myocardial level should be considered when interpreting or comparing mapping values in veterinary cardiac MRI. Future canine mapping studies should report segment availability, use consistent slice prescription, apply systematic frequency scouting and localized cardiac shimming, and consider practical strategies to shift residual bSSFP banding away from the LV myocardium when possible, such as careful center-frequency selection and heart-focused shim adjustment. Sequence choices less sensitive to off-resonance effects, body-size-adapted slice prescription, and post-processing strategies that improve native/post-contrast registration may further reduce partial-volume effects and ECV variability.

### 4.4 Comparison with prior literature and methodological implications

At 3.0 T, mean rest-global LV myocardial values in this canine cohort were 939.01 ms for T1 native and 44.46 ms for T2 (Table 2). In healthy humans at 3.0 T, T1 native values typically range from 1,150 to 1,180 ms and T2 from 44 to 47 ms, indicating that canine T2 values fall within a comparable range, whereas T1 native values are lower (19). A recent 1.5-T canine mapping study reported higher T1 native (984–1,013 ms) and T2 (54–65 ms) values (21). Such differences align with known dependencies of mapping parameters on field strength, sequence design, vendor, and post-processing methods (4, 31). Accordingly, absolute myocardial mapping values in dogs should be interpreted as protocol-specific preliminary estimates obtained under the present 3.0 T anesthetic and imaging protocol rather than as definitive reference values or as directly comparable values across species or imaging systems.

Stress–rest reactivity may provide physiologic information beyond absolute mapping values alone. In human studies, adenosine stress T1 reactivity is typically reported at approximately 4–8% in non-ischemic myocardium, whereas infarcted or ischemic myocardium may show elevated resting T1 with blunted or absent stress responses (9, 32, 33). The 6.7% global T1 native increase observed in the present canine cohort falls within this reported human range, supporting the physiologic relevance of the standardized adenosine stress protocol (Table 2). Because direct myocardial blood flow was not measured and predefined stress-adequacy thresholds were not applied, this finding should be interpreted as a protocol-specific stress-associated mapping response rather than as proof of preserved vasodilator reserve in each dog. Level-wise differences were also observed in selected parameter–condition combinations (Table 5), reinforcing the need to consider sampling level when comparing canine myocardial mapping values. Collectively, these findings support non-contrast adenosine stress T1 reactivity as a hypothesis-generating baseline measure for future studies comparing clinically asymptomatic and diseased canine myocardium, while the observed basal–apical gradients should be interpreted as protocol- and level-dependent variation rather than disease-specific patterns.

Adenosine stress also elicited a measurable T2 response (Table 2). In humans, myocardial T2 can increase during adenosine stress, reflecting changes in myocardial perfusion and blood volume, and experimental studies support myocardial blood flow as an important determinant of this response (34). These observations are consistent with T2 sensitivity to pharmacologic vasodilator stress at 3.0 T and suggest that T2 may provide complementary information to T1 mapping.

ECV remained stable across stress and rest and was approximately 21% at the global level, which is lower than published human 3.0 T values of approximately 26–27% (35, 36). This comparison should be interpreted cautiously because ECV is influenced by species, field strength, pulse sequence, hematocrit measurement, contrast timing, and post-processing methods. The minimal stress–rest difference aligns with expectations that vasodilator stress does not acutely alter extracellular space fraction. This stability suggests that ECV may serve as a relatively stable structural comparator for interpreting stress-associated changes in T1 native and T2, although this interpretation remains protocol-specific and requires validation in larger canine cohorts.

Overall, these findings support adenosine stress–rest mapping at 3.0 T in anesthetized dogs as an initial methodological step, provide protocol-specific preliminary level-wise estimates for T1, T2, and ECV, and suggest that non-contrast stress T1/T2 reactivity warrants further investigation in veterinary myocardial assessment (9, 33).

### 4.5 Limitations

A key limitation is the small sample size, largely driven by owner consent and the need for general anesthesia, which constrained enrollment. As a result, statistical power to identify small differences, especially in the 16 myocardial segments, was limited, and the three-slice design may not fully capture regional heterogeneity across all myocardial territories. However, the study focused on a prospectively enrolled, well-characterized cohort scanned using a standardized 3.0 T stress–rest mapping protocol, thereby providing rare, systematically acquired protocol-specific preliminary data for canine myocardial mapping despite the relatively small animal population.

A further limitation is physiologic variability associated with anesthesia and heart-rate variation. Butorphanol, propofol, and isoflurane were necessary to ensure immobility, controlled ventilation, and safe cardiac MRI acquisition in client-owned dogs; however, these agents may influence arterial pressure, cardiac output, myocardial contractility, heart rate, and vasodilator responsiveness (37–39). Although the same anesthetic protocol was used for both sessions, rest and stress examinations were performed on different days, and small differences in physiologic state may have influenced myocardial relaxation times and derived mapping values. Arterial blood gas and lactate measurements were not obtained; therefore, potential differences in acid–base status, arterial oxygenation, arterial carbon dioxide tension, or metabolic state could not be directly assessed. In addition, MOLLI-based T1 mapping can be affected by heart rate because incomplete longitudinal recovery between inversion pulses may influence T1 estimation at higher heart rates (24). Heart rate varied among dogs and between stress and rest sessions, heart-rate correction was not applied, and exploratory analyses showed positive associations of heart rate with T1 native and T2. Therefore, the study design could not separate direct heart-rate effects, anesthetic effects, and the broader adenosine stress response. Future canine stress-mapping studies should incorporate stricter physiologic standardization, detailed anesthetic documentation, arterial blood gas monitoring when feasible, and heart-rate-aware or heart-rate-corrected mapping approaches.

Although intraobserver repeatability of segment-level ROI measurements was high, interobserver reproducibility was not assessed. Therefore, the reported protocol-specific mapping estimates may still be influenced by observer-dependent ROI placement, and future studies should include multi-observer reproducibility testing.

Adenosine stress adequacy is another important limitation. The 140 μg/kg/min infusion rate and the ≥5-min delay before stress mapping were selected to be consistent with prior vasodilator stress CMR protocols and canine adenosine stress perfusion CMR studies (16, 23). However, quantitative myocardial blood flow was not measured, and no predefined hemodynamic threshold was used to confirm adequate vasodilation in each dog. Therefore, the observed stress–rest differences should be interpreted as protocol-specific adenosine-associated mapping responses rather than as direct measures of quantitatively confirmed hyperemia. Future studies should incorporate direct perfusion quantification or predefined stress-adequacy criteria when feasible.

Despite frequency scouting and heart-restricted shimming (Figure 3), residual off-resonance banding and map-quality degradation still affected segment availability. In the segment-level audit, 23 of 896 parameter-specific segment observations (2.6%) were excluded because the final analyzable ROI pixel count was zero. Exclusions were uncommon overall and were not confined to a single ventricular level, although they were relatively more frequent for ECV under the rest condition. This likely reflects the additional dependence of ECV on accurate registration between native and post-contrast T1 maps, as well as susceptibility to boundary mismatch and contour misregistration. Therefore, although segment exclusion was limited in extent, a small degree of level- or region-specific bias cannot be fully excluded, especially for regional ECV estimates. These technical considerations should be incorporated into future canine 3.0 T mapping protocols to improve regional data completeness and reduce protocol-dependent variability.

Finally, enrollment was limited to clinically asymptomatic dogs without imaging evidence of myocardial ischemia. Although some dogs had trivial-to-mild valvular regurgitation without echocardiographic remodeling, regurgitation-related myocardial effects cannot be entirely excluded. Similarly, mild clinically stable non-cardiac abnormalities, such as hepatic enzyme elevation, were not considered exclusionary but may limit the interpretation of the cohort as strictly healthy. Accordingly, the reported values should be interpreted as protocol-specific preliminary estimates from clinically asymptomatic, non-ischemic, near-normal/subclinical dogs rather than as definitive healthy reference intervals. Future studies should include defined ischemic/inflammatory/remodeling phenotypes to establish disease-specific stress- and level-dependent patterns.

Despite these limitations, the current results indicate that adenosine stress–rest myocardial mapping at 3.0 T can be performed under general anesthesia in dogs and provide a methodological basis for future veterinary and translational studies. Clinical translation will require larger cohorts, defined disease groups, direct assessment of stress adequacy, and further reproducibility validation.

## 5 Conclusion

At 3.0 T, quantitative cardiac MRI enables assessment of myocardial tissue properties in dogs. Adenosine stress was associated with higher global T1 native and T2 compared with rest, whereas no significant stress–rest changes were detected in T1 post or ECV. Mapping values also varied according to basal, mid, and apical short-axis levels, with basal predominance for T1 native, a modest stress-only basal–mid difference for ECV, and lower basal T2 at rest. These preliminary findings suggest that non-contrast quantification of adenosine-associated myocardial responses is technically achievable in anesthetized dogs and indicate that myocardial level should be considered when interpreting or comparing myocardial mapping indices. These results provide protocol-specific preliminary estimates for adenosine stress myocardial mapping at 3.0 T in anesthetized dogs and support further investigation before clinical application in veterinary patients.

## Data Availability

The original contributions presented in the study are included in the article/Supplementary material, further inquiries can be directed to the corresponding author.

## Glossary

AHA: American Heart Association
B0: Static magnetic field
B1: Radiofrequency field
BP: Blood pressure
bSSFP: Balanced steady-state free precession
CMR: Cardiovascular magnetic resonance
DIA: Diastolic blood pressure
dz: Standardized paired effect size (Cohen’s dz)
ECG: Electrocardiography
ECV: Extracellular volume fraction
GEE: Generalized estimating equations
HR: Heart rate
IACUC: Institutional Animal Care and Use Committee
IV: Intravenous
LA: Left atrium
LGE: Late gadolinium enhancement
LV: Left ventricle
MAP: Mean arterial pressure
MOLLI: Modified Look–Locker inversion recovery
MR: Mitral regurgitation
MRI: Magnetic resonance imaging
RA: Right atrium
ROI: Region of interest
RV: Right ventricle
SYS: Systolic blood pressure
T1: Longitudinal relaxation time
T2: Transverse relaxation time
TR: Tricuspid regurgitation
TTE: Transthoracic echocardiography

## References

[1] Abbara S Blanke P Maroules CD Cheezum M Choi AD Han BK . SCCT guidelines for the performance and acquisition of coronary computed tomographic angiography: a report of the society of cardiovascular computed tomography guidelines committee: endorsed by the north American Society for Cardiovascular Imaging (NASCI). J Cardiovasc Comput Tomogr. (2016) 10:435–49. doi: 10.1016/j.jcct.2016.10.002, 27780758

[2] Dilsizian V Bacharach SL Beanlands RS Bergmann SR Delbeke D Dorbala S . ASNC imaging guidelines/SNMMI procedure standard for positron emission tomography (PET) nuclear cardiology procedures. J Nucl Cardiol. (2016) 23:1187–226. doi: 10.1007/s12350-016-0522-3, 27392702

[3] Gilbert SH McConnell FJ Holden AV Sivananthan MU Dukes-McEwan J. The potential role of MRI in veterinary clinical cardiology. Vet J. (2010) 183:124–34. doi: 10.1016/j.tvjl.2008.11.018, 19136284

[4] Messroghli DR Moon JC Ferreira VM Grosse-Wortmann L He T Kellman P . Clinical recommendations for cardiovascular magnetic resonance mapping of T1, T2, T2* and extracellular volume: a consensus statement by the Society for Cardiovascular Magnetic Resonance (SCMR) endorsed by the European Association for Cardiovascular Imaging (EACVI). J Cardiovasc Magn Reson. (2017) 19:75. doi: 10.1186/s12968-017-0389-8, 28992817 PMC5633041

[5] Fries RC. Current use of cardiac MRI in animals. J Vet Cardiol. (2024) 51:13–23. doi: 10.1016/j.jvc.2023.11.006, 38052149

[6] Kramer CM Barkhausen J Bucciarelli-Ducci C Flamm SD Kim RJ Nagel E. Standardized cardiovascular magnetic resonance imaging (CMR) protocols: 2020 update. J Cardiovasc Magn Reson. (2020) 22:17. doi: 10.1186/s12968-020-00607-1, 32089132 PMC7038611

[7] Jeong H Lee H Jung J Kim H Yu J Yoon H . Evaluation of left ventricular function with cardiac magnetic resonance imaging and echocardiography after administration of dobutamine and esmolol in healthy beagle dogs. J Vet Med Sci. (2021) 83:581–91. doi: 10.1292/jvms.18-0703 33473057 PMC8111355

[8] O'Brien AT Gil KE Varghese J Simonetti OP Zareba KM. T2 mapping in myocardial disease: a comprehensive review. J Cardiovasc Magn Reson. (2022) 24:33. doi: 10.1186/s12968-022-00866-0, 35659266 PMC9167641

[9] Liu A Wijesurendra RS Francis JM Robson MD Neubauer S Piechnik SK . Adenosine stress and rest T1 mapping can differentiate between ischemic, infarcted, remote, and Normal myocardium without the need for gadolinium contrast agents. JACC Cardiovasc Imaging. (2016) 9:27–36. doi: 10.1016/j.jcmg.2015.08.018, 26684978 PMC4708879

[10] Keene BW Atkins CE Bonagura JD Fox PR Häggström J Fuentes VL . ACVIM consensus guidelines for the diagnosis and treatment of myxomatous mitral valve disease in dogs. J Vet Intern Med. (2019) 33:1127–40. doi: 10.1111/jvim.15488, 30974015 PMC6524084

[11] Borgarelli M Buchanan JW. Historical review, epidemiology and natural history of degenerative mitral valve disease. J Vet Cardiol. (2012) 14:93–101. doi: 10.1016/j.jvc.2012.01.011, 22386588

[12] Herman E Eldridge S. Spontaneously occurring cardiovascular lesions in commonly used laboratory animals. Cardiooncology. (2019) 5:6. doi: 10.1186/s40959-019-0040-y, 32154013 PMC7048038

[13] Falk T Ljungvall I Zois NE Höglund K Olsen LH Pedersen HD . Cardiac troponin-I concentration, myocardial arteriosclerosis, and fibrosis in dogs with congestive heart failure because of myxomatous mitral valve disease. J Vet Intern Med. (2013) 27:500–6. doi: 10.1111/jvim.12075, 23551840

[14] Layland J Carrick D Lee M Oldroyd K Berry C. Adenosine: physiology, pharmacology, and clinical applications. JACC Cardiovasc Interv. (2014) 7:581–91. doi: 10.1016/j.jcin.2014.02.009, 24835328

[15] Clark WA Winter RL Aarnes TK Green EM Mikrut K Ruz P . Utility of cardiac MRI to diagnose myocardial ischemia and fibrosis in dogs with cardiomegaly secondary to myxomatous mitral valve disease. Am J Vet Res. (2022) 83:1–10. doi: 10.2460/ajvr.22.05.0076, 35905145

[16] Richter H Kircher PR Joerger FB Bruellmann E Dennler M. Assessment of myocardial perfusion at rest and during stress using dynamic first-pass contrast-enhanced magnetic resonance imaging in healthy dogs. Front Vet Sci. (2018) 5:211. doi: 10.3389/fvets.2018.00211, 30234137 PMC6131641

[17] Cerqueira MD Weissman NJ Dilsizian V Jacobs AK Kaul S Laskey WK . Standardized myocardial segmentation and nomenclature for tomographic imaging of the heart. a statement for healthcare professionals from the cardiac imaging Committee of the Council on clinical cardiology of the American Heart Association. Circulation. (2002) 105:539–42. doi: 10.1161/hc0402.102975, 11815441

[18] Rodríguez-Padilla J Petras A Magat J Bayer J Bihan-Poudec Y El Hamrani D . Impact of intraventricular septal fiber orientation on cardiac electromechanical function. Am J Physiol Heart Circ Physiol. (2022) 322:H936–52. doi: 10.1152/ajpheart.00050.2022, 35302879 PMC9109800

[19] von Knobelsdorff-Brenkenhoff F Prothmann M Dieringer MA Wassmuth R Greiser A Schwenke C . Myocardial T1 and T2 mapping at 3 T: reference values, influencing factors and implications. J Cardiovasc Magn Reson. (2013) 15:53. doi: 10.1186/1532-429x-15-53, 23777327 PMC3702448

[20] Xu Z Li W Wang J Wang F Sun B Xiang S . Reference ranges of myocardial T1 and T2 mapping in healthy Chinese adults: a multicenter 3T cardiovascular magnetic resonance study. J Cardiovasc Magn Reson. (2023) 25:64. doi: 10.1186/s12968-023-00974-5, 37968645 PMC10652608

[21] Yun D Lee HW Jin W Lee K Lee SK. Multiparametric myocardial mapping using cardiac magnetic resonance imaging in healthy dogs: reproducibility, repeatability, and differences across slices, segments, and sequences. Vet Radiol Ultrasound. (2024) 65:628–39. doi: 10.1111/vru.13406, 38958215

[22] Dennler M Baron Toaldo M Makara M Lautenschlager IE Ribbers G Wang-Leandro A . Recommendations for standardized plane definition in canine cardiac MRI. Vet Radiol Ultrasound. (2020) 61:696–704. doi: 10.1111/vru.12911 32996225

[23] Kotecha T Martinez-Naharro A Boldrini M Knight D Hawkins P Kalra S . Automated pixel-wise quantitative myocardial perfusion mapping by CMR to detect obstructive coronary artery disease and coronary microvascular dysfunction: validation against invasive coronary physiology. JACC Cardiovasc Imaging. (2019) 12:1958–69. doi: 10.1016/j.jcmg.2018.12.022, 30772231 PMC8414332

[24] Kellman P Hansen MS. T1-mapping in the heart: accuracy and precision. J Cardiovasc Magn Reson. (2014) 16:2. doi: 10.1186/1532-429x-16-2, 24387626 PMC3927683

[25] Shah SA Reagan CE French BA Epstein FH. Molecular mechanisms of adenosine stress T1 mapping. Circ Cardiovasc Imaging. (2021) 14:e011774. doi: 10.1161/circimaging.120.011774, 33706537 PMC7969455

[26] Oshinski JN Delfino JG Sharma P Gharib AM Pettigrew RI. Cardiovascular magnetic resonance at 3.0 T: current state of the art. J Cardiovasc Magn Reson. (2010) 12:55. doi: 10.1186/1532-429x-12-55, 20929538 PMC2964699

[27] Rajiah P Bolen MA. Cardiovascular MR imaging at 3 T: opportunities, challenges, and solutions. Radiographics. (2014) 34:1612–35. doi: 10.1148/rg.346140048, 25310420

[28] Mahmod M Piechnik SK Levelt E Ferreira VM Francis JM Lewis A . Adenosine stress native T1 mapping in severe aortic stenosis: evidence for a role of the intravascular compartment on myocardial T1 values. J Cardiovasc Magn Reson. (2014) 16:92. doi: 10.1186/s12968-014-0092-y, 25410203 PMC4237748

[29] Weyers JJ Ramanan V Javed A Barry J Larsen M Nayak K . Myocardial blood flow is the dominant factor influencing cardiac magnetic resonance adenosine stress T2. NMR Biomed. (2022) 35:e4643. doi: 10.1002/nbm.4643, 34791720 PMC8828684

[30] Baeßler B Schaarschmidt F Stehning C Schnackenburg B Maintz D Bunck AC. A systematic evaluation of three different cardiac T2-mapping sequences at 1.5 and 3T in healthy volunteers. Eur J Radiol. (2015) 84:2161–70. doi: 10.1016/j.ejrad.2015.08.002, 26276731

[31] Fenski M Gröschel J Gatehouse P Kolbitsch C Schulz-Menger J. Artifacts in cardiac T1 and T2 mapping techniques - influence on reliable quantification. J Cardiovasc Magn Reson. (2025) 27:101934. doi: 10.1016/j.jocmr.2025.101934, 40721019 PMC12670910

[32] Burrage MK Shanmuganathan M Zhang Q Hann E Popescu IA Soundarajan R . Cardiac stress T1-mapping response and extracellular volume stability of MOLLI-based T1-mapping methods. Sci Rep. (2021) 11:13568. doi: 10.1038/s41598-021-92923-4, 34193894 PMC8245629

[33] Levelt E Piechnik SK Liu A Wijesurendra RS Mahmod M Ariga R . Adenosine stress CMR T1-mapping detects early microvascular dysfunction in patients with type 2 diabetes mellitus without obstructive coronary artery disease. J Cardiovasc Magn Reson. (2017) 19:81. doi: 10.1186/s12968-017-0397-8, 29070069 PMC5655826

[34] Nickander J Themudo R Thalen S Sigfridsson A Xue H Kellman P . The relative contributions of myocardial perfusion, blood volume and extracellular volume to native T1 and native T2 at rest and during adenosine stress in normal physiology. J Cardiovasc Magn Reson. (2019) 21:73. doi: 10.1186/s12968-019-0585-9 31767018 PMC6876099

[35] Kositanurit W Theerasuwipakorn N Vorasettakarnkij Y Ponkanist K Lerdkhonsan C Tumkosit M . Reference values of myocardial native T1 and extracellular volume in patients without structural heart disease and had negative 3T cardiac magnetic resonance adenosine stress test. Int J Cardiol Heart Vasc. (2023) 45:101181. doi: 10.1016/j.ijcha.2023.101181, 36793331 PMC9923153

[36] Lee JJ Liu S Nacif MS Ugander M Han J Kawel N . Myocardial T1 and extracellular volume fraction mapping at 3 tesla. J Cardiovasc Magn Reson. (2011) 13:75. doi: 10.1186/1532-429x-13-75, 22123333 PMC3269374

[37] Cattai A Rabozzi R Ferasin H Isola M Franci P. Haemodynamic changes during propofol induction in dogs: new findings and approach of monitoring. BMC Vet Res. (2018) 14:282. doi: 10.1186/s12917-018-1608-8, 30208893 PMC6134702

[38] Conzen PF Hobbhahn J Goetz AE Habazettl H Granetzny T Peter K . Myocardial contractility, blood flow, and oxygen consumption in healthy dogs during anesthesia with isoflurane or enflurane. J Cardiothorac Anesth. (1989) 3:70–7. doi: 10.1016/0888-6296(89)90014-8, 2520643

[39] Trim CM. Cardiopulmonary effects of butorphanol tartrate in dogs. Am J Vet Res. (1983) 44:329–31. doi: 10.2460/ajvr.1983.44.02.329, 6830022

