# Supplementary material for: Quantitative myocardial mapping using 3.0 T cardiac magnetic resonance imaging in dogs: adenosine stress–rest evaluation
Source: Front Vet Sci. 2026 Jul 15;13:1850159. doi: 10.3389/fvets.2026.1850159 (PMC13414728; doi:10.3389/fvets.2026.1850159)
Supplement: Supplementary file 1 [file Table_1.DOCX]

# Supplementary Material

Supplementary Table 1. Median [IQR] values and Shapiro–Wilk normality results for paired stress–rest differences in myocardial mapping parameters

| **Parameter** | **Global/Level** | **Stress median [IQR]** | **Rest median [IQR]** | **Δ median [IQR]** | **Δ% median [IQR]** | **Shapiro–Wilk p-value for paired Δ** |
| --- | --- | --- | --- | --- | --- | --- |
| T1 native | Global | 1022.45 [986.10–1034.29] | 950.13 [901.57–974.59] | 55.05 [43.84–74.88] | 6.01 [4.42–8.19] | 0.110 |
| T1 native | Basal | 1035.21 [998.68–1059.97] | 942.55 [916.44–993.15] | 72.11 [63.61–93.64] | 7.24 [6.54–10.33] | 0.815 |
| T1 native | Mid | 1000.41 [966.72–1020.75] | 953.79 [893.92–959.07] | 34.40 [33.14–51.17] | 3.76 [3.47–5.45] | 0.001 |
| T1 native | Apical | 991.78 [973.33–1011.66] | 966.02 [890.91–971.31] | 38.48 [25.02–82.35] | 3.89 [2.74–8.69] | 0.153 |
| T1 post | Global | 573.63 [523.76–631.93] | 595.56 [576.79–637.70] | −55.18 [−113.94–26.32] | −8.07 [−17.88–4.65] | 0.152 |
| T1 post | Basal | 583.15 [513.94–631.89] | 601.30 [583.51–627.12] | −57.89 [−113.18–25.27] | −8.48 [−18.07–4.42] | 0.385 |
| T1 post | Mid | 564.84 [526.26–629.51] | 578.69 [574.30–642.81] | −60.09 [−114.22–30.15] | −8.75 [−18.12–5.27] | 0.155 |
| T1 post | Apical | 568.79 [537.17–630.89] | 608.02 [571.32–648.87] | −52.25 [−105.46–16.99] | −7.65 [−17.03–3.15] | 0.090 |
| ECV | Global | 20.84 [19.91–21.86] | 19.83 [19.37–20.55] | 0.45 [−0.71–1.49] | 2.21 [−3.10–7.84] | 0.417 |
| ECV | Basal | 21.68 [20.11–22.66] | 19.88 [18.96–20.80] | 1.12 [−1.05–2.12] | 5.43 [−4.41–11.36] | 0.414 |
| ECV | Mid | 20.17 [19.77–21.54] | 20.30 [19.51–20.44] | −0.14 [−0.57–0.97] | −0.67 [−2.36–5.14] | 0.157 |
| ECV | Apical | 20.68 [19.79–21.17] | 19.96 [19.38–20.43] | 0.48 [−0.60–1.26] | 2.38 [−3.00–6.49] | 0.588 |
| T2 | Global | 49.91 [45.41–51.96] | 45.53 [43.37–46.21] | 4.21 [2.66–6.40] | 9.03 [5.89–14.96] | 0.413 |
| T2 | Basal | 45.49 [44.46–49.50] | 42.36 [41.32–43.95] | 3.83 [1.68–6.72] | 9.34 [3.74–16.13] | 0.150 |
| T2 | Mid | 49.60 [46.20–52.37] | 46.14 [45.14–47.02] | 3.02 [1.85–5.57] | 6.48 [3.93–12.60] | 0.139 |
| T2 | Apical | 50.73 [46.50–51.90] | 46.74 [43.49–47.76] | 4.00 [2.77–6.46] | 8.55 [6.36–15.84] | 0.132 |

Values are median [interquartile range] across dogs (n = 7). Δ indicates the paired difference calculated as stress − rest. Δ% indicates the percentage change relative to rest. Shapiro–Wilk p-values were calculated for paired stress–rest differences. ECV values are expressed as percentages; T1 native, T1 post, and T2 values are expressed in milliseconds.

Supplementary Table 2. Exploratory partial Spearman correlations between heart rate and myocardial mapping parameters

| **Parameter** | **r** | **p-value** |
| --- | --- | --- |
| T1 native | 0.675 | 0.008 |
| T1 post | −0.431 | 0.124 |
| ECV | −0.218 | 0.454 |
| T2 | 0.548 | 0.043 |

Correlation coefficients (r) and p-values were obtained from exploratory partial Spearman correlation analyses between heart rate and myocardial mapping parameters. These analyses were performed to evaluate heart rate as a potential physiologic covariate. ECV, extracellular volume fraction.

Supplementary Table 3. Summary of myocardial segment exclusions during quantitative mapping analysis

| **Parameter** | **Condition** | **Total possible segments** | **Excluded segments** | **Exclusion rate (%)** | **Basal** | **Mid** | **Apical** |
| --- | --- | --- | --- | --- | --- | --- | --- |
| T1 native | Stress | 112 | 3 | 2.7 | 1 | 1 | 1 |
| T1 native | Rest | 112 | 4 | 3.6 | 1 | 1 | 2 |
| T1 post | Stress | 112 | 1 | 0.9 | 0 | 1 | 0 |
| T1 post | Rest | 112 | 0 | 0.0 | 0 | 0 | 0 |
| ECV | Stress | 112 | 2 | 1.8 | 1 | 1 | 0 |
| ECV | Rest | 112 | 11 | 9.8 | 3 | 4 | 4 |
| T2 | Stress | 112 | 2 | 1.8 | 1 | 1 | 0 |
| T2 | Rest | 112 | 0 | 0.0 | 0 | 0 | 0 |
| Total | Stress | 448 | 8 | 1.8 | 3 | 4 | 1 |
| Total | Rest | 448 | 15 | 3.3 | 4 | 5 | 6 |
| **Overall** | **Stress + Rest** | **896** | **23** | **2.6** | **7** | **9** | **7** |

Excluded segments were defined as myocardial segments with a final analyzable ROI pixel count of zero in the segment-level dataset. Basal, mid, and apical levels contained 6, 6, and 4 AHA segments per dog, respectively. Across all dogs, conditions, parameters, and myocardial levels, 23 of 896 parameter-specific segment observations were excluded. Exclusions were uncommon overall and were not confined to a single ventricular level, although ECV under the rest condition showed the highest exclusion frequency.

Supplementary Table 4. Intraobserver repeatability of segment-level myocardial mapping measurements

| **Parameter** | **Paired segments** | **ICC** | **95% CI** | **CV (%)** |
| --- | --- | --- | --- | --- |
| T1 native | 217 | 0.978 | 0.974–0.982 | 1.0 |
| T1 post | 223 | 0.984 | 0.980–0.986 | 1.4 |
| ECV | 211 | 0.917 | 0.895–0.931 | 3.6 |
| T2 | 222 | 0.962 | 0.952–0.968 | 2.2 |

Intraobserver repeatability was assessed using segment-level paired measurements from the first and repeated ROI analyses. Repeated ROI delineation was performed by the same observer after a 5-month washout interval while blinded to the first measurements. Segments with zero analyzable ROI pixels were excluded from the paired analysis. ICCs were calculated using a two-way mixed-effects absolute-agreement model for single measurements. CV was calculated as the within-observer coefficient of variation.
